# Recent Advances in Benzimidazole–Triazole Hybrids for Single- and Multi-Target Protein Kinase Inhibition

**DOI:** 10.3390/ph19040623

**Published:** 2026-04-15

**Authors:** Hamzeh M. Abu Al Rub, Ahmed G. Eissa

**Affiliations:** 1 College of Pharmacy, Al Ain University, Abu Dhabi 64141, United Arab Emirates; 202320736@aau.ac.ae (H.M.A.A.R.); ahmed.eissa@aau.ac.ae (A.G.E.); 2 Department of Medicinal Chemistry, Faculty of Pharmacy, Zagazig University, Zagazig 44519, Egypt

**Keywords:** Benzimidazole, triazole, heterocyclic scaffolds, kinase inhibitors, multitarget, anticancer

## Abstract

Background/Objectives: Protein kinases play a crucial role in cancer initiation, progression, and therapeutic resistance by regulating signalling pathways involved in tumour growth and survival. Consequently, they represent major targets in anticancer drug discovery. Among heterocyclic scaffolds explored in kinase inhibitor design, benzimidazole has emerged as a privileged structure due to its strong hydrogen-bonding capability and structural resemblance to purine moieties. Triazole motifs are also widely incorporated into bioactive molecules because of their metabolic stability, favourable electronic properties, and ability to establish key interactions within kinase active sites. This review aims to summarise and critically discuss benzimidazole- and triazole-based kinase inhibitors, both as individual scaffolds and as hybrid systems, with emphasis on their kinase targets and multitarget potential. Methods: The relevant literature was surveyed from major scientific databases focusing on studies describing the synthesis, biological evaluation, and molecular modelling of benzimidazole- and triazole-containing kinase inhibitors. Results: Numerous studies demonstrate that both benzimidazole and triazole scaffolds exhibit significant kinase inhibitory activity against oncogenic targets, including EGFR, cyclin-dependent kinases (CDKs), and components of the PI3K/Akt/mTOR signalling pathway. Hybrid molecules combining these pharmacophores frequently enhance binding interactions and facilitate the development of multitarget kinase inhibitors. Structure–activity relationship trends indicate that pharmacophore accessibility, substitution patterns, and linker architecture influence inhibitory potency and selectivity. Conclusions: Overall, benzimidazole- and triazole-based scaffolds represent promising platforms for developing next-generation multitarget anticancer agents and provide valuable insights for the rational design of improved kinase inhibitors.

## 1. Introduction

**Figure 1:**
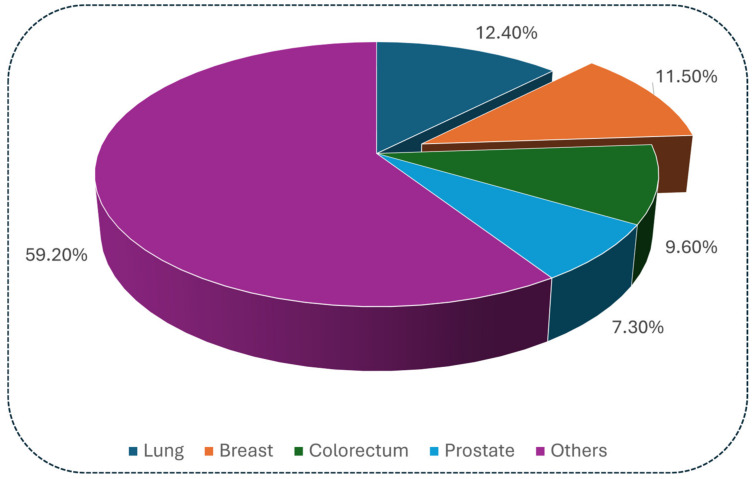
Global cancer incidence by cancer type based on GLOBOCAN estimates [2].

Cancer continues to rank among the leading causes of mortality worldwide in 2025. According to recent global estimates, around 18.5 million new cancer cases and 10.4 million cancer-related deaths occurred in 2023 [1]. According to GLOBOCAN [2], in 2022, lung cancer accounted for the highest number of incidences, representing 12.4% of all cases, followed by breast cancer at 11.5%, and colorectal cancer at 9.6% (Figure 1).

Alarmingly, projections indicate global cancer incidence could surpass 35 million new cases by 2050, marking a 77% rise compared to the estimated 20 million cases recorded in 2022 [3]. Despite significant advances in the development of chemotherapeutics and targeted therapies, therapeutic resistance remains a major clinical challenge, which leads to the ineffectiveness of current chemotherapeutic agents and thus limits long-term treatment success and patient survival [4].

Common mechanisms of action for chemotherapeutics include enzyme inhibition, transcriptional regulation, angiogenesis suppression, DNA groove binding, gene expression modulation, DNA intercalation, inhibition of DNA replication, and microtubule disruption [5]. Despite the broad spectrum of activity exhibited by conventional cytotoxic drugs, their nonspecific nature often damages healthy cells alongside tumour cells. This limitation has shifted research toward targeted strategies such as kinase inhibition, which enable more precise control of cancer cell progression and survival [6].

Heterocyclic scaffolds form the foundation of many approved anticancer drugs, especially kinase inhibitors, because they can mimic ATP and bind effectively to the hinge region of kinases. Well-known drugs such as Erlotinib, Gefitinib, and Lapatinib contain these structures, which enable important hydrogen-bonding interactions and improve binding within the ATP-binding site. Their clinical success demonstrates the value of heterocyclic systems in drug design and supports continued exploration of such scaffolds.

Recent advances in our understanding of the fundamental molecular mechanisms underlying cancer cell signalling have elucidated a crucial role for kinases in the carcinogenesis and metastases of various types of cancer [7,8].

## 2. Key/Crucial Kinases in Cancer

Several kinases are involved in tumour cell transformation and survival. A key kinase family that plays a vital role in tumour proliferative process is the phosphoinositide 3-kinase (PI3K) family, which is commonly mutated in cancer [9]. Of this family, PI3KCA enzyme catalyses the conversion of membrane phospholipids into phosphatidylinositol-3,4,5-trisphosphate (PIP_3_), a pivotal second messenger that activates downstream effectors such as protein kinase AKT. Through this signalling cascade, the PI3K/Akt pathway regulates the cell cycle by modulating its downstream targets, thereby promoting tumour cell growth, proliferation, and survival. Hence, it is a frequent target in cancer management to suppress malignant progression. However, drug resistance remains a major obstacle to effective treatment and often contributes to tumour recurrence, with dysregulated PI3K/Akt signalling closely linked to the development of such resistance [10,11]. Aurora-A is a kinase found to be amplified in several cases of ovarian cancer [12,13,14]. It phosphorylates the tumour suppressor p53 at Ser215, thereby hindering its DNA-binding ability and impairing normal cell cycle checkpoint control [14]. Another example of tumour-related kinases is mammalian target of rapamycin (mTOR). The mTOR pathway is deeply involved in numerous fundamental cellular functions, including regulating growth and division, maintaining cell viability, controlling autophagic activity, coordinating metabolic processes, and influencing immune system responses [15,16]. The dysregulated activation of mTOR in human cancers can result from activating mutations within the mTOR pathway, amplification or overexpression of components of the mTOR complexes, as well as mutations or loss of its negative regulators [17]. Since mTOR is deeply involved in driving tumour development and progression, targeting this pathway with specific inhibitors has emerged as a promising therapeutic approach in cancer treatment. Another crucial family of kinases implicated in cancer progression is the cyclin-dependent kinases (CDKs). CDKs regulate cell cycle transitions and other essential cellular functions, including transcription [18]. Dysregulation of CDK is frequently observed across a wide range of human cancers, contributing to uncontrolled proliferation and tumour progression [19]. Building on this, epidermal growth factor receptor (EGFR) is another key kinase that possesses a critical role in regulating cell proliferation, differentiation, and survival. Mutations or overexpression of EGFR are strongly associated with the development and progression of several human cancers [20]. Given their extensive involvement in various stages of tumour development and progression, EGFR and its family members have become key targets for cancer therapies [21].

## 3. Common Heterocyclic Scaffolds in Anticancer Drug Design

Numerous structural frameworks have been explored and optimised as kinase inhibitors, with certain heterocyclic cores consistently emerging as privileged motifs in anticancer drug design. Among them, benzimidazole [22], quinazoline [23], pyrazole [24], thiazole [25], imidazopyridine [26], pyrimidine [27], and triazole [28] scaffolds have been extensively investigated for their ability to modulate kinase activity in various cancer types. This review will focus primarily on recent advancements in benzimidazole and triazole-based derivatives, given their rising prominence and potential in the development of next-generation anticancer agents.

Benzimidazoles represent a prominent class of fused heterocyclic compounds, structurally characterised by the fusion of a benzene ring with an imidazole moiety at the 4 and 5 positions. The term *benzimidazole* denotes the unsubstituted parent scaffold (C_7_H_6_N_2_), whereas benzimidazoles broadly refers to its derivatives bearing various substituents at different positions on the core ring system. The benzimidazole scaffold has emerged as a privileged pharmacophore in anticancer drug discovery owing to its broad-spectrum cytotoxic activity and diverse mechanisms of tumour inhibition, including microtubule disruption and kinase inhibition. Numerous FDA-approved drugs incorporate benzimidazole moieties [29,30,31,32] (Table 1).

Benzimidazoles can be synthesised via several well-established methods, including condensation reactions of *o*-phenylenediamine with a variety of carbonyl compounds under both acidic and catalytic conditions [33]. One notable approach involves the acid-mediated condensation of *o*-phenylenediamine with carboxylic acids under reflux [34].

**Table 1:** Some FDA-approved anti-cancer compounds containing benzimidazole.

Compound Name	Structure	Cancer Type	Reference
Abemaciclib	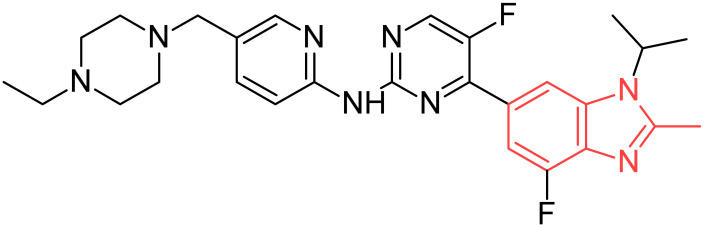	Advanced or metastatic breast cancers.	[35]
Bendamustine	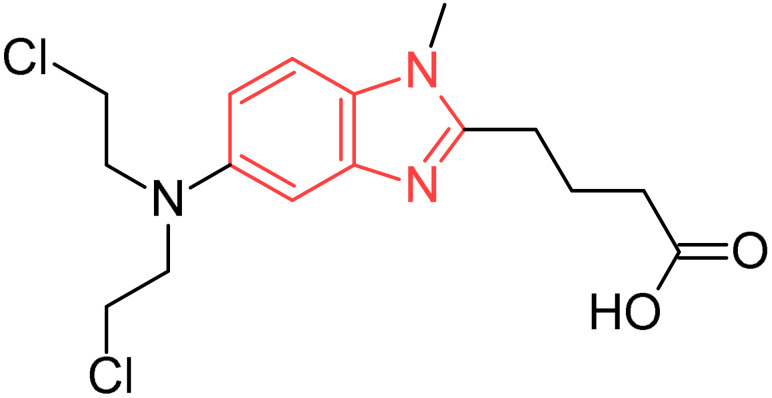	Lymphocytic leukaemia	[30]
Binimetinib	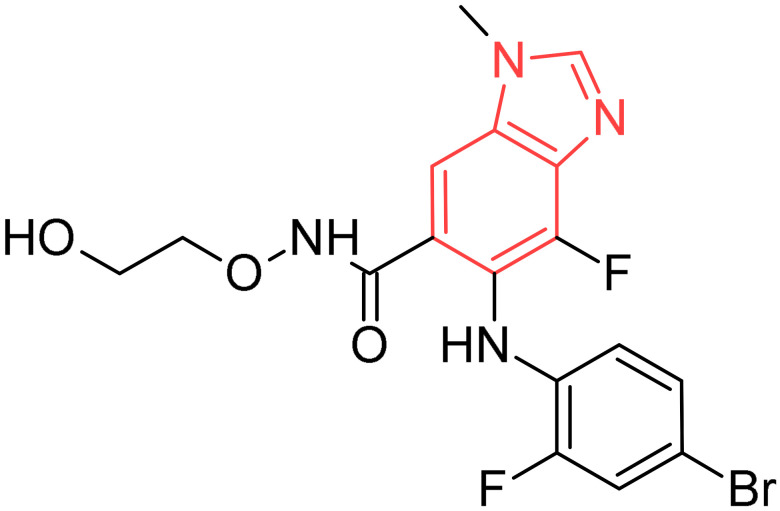	Metastatic melanoma	[29]
Selumetinib	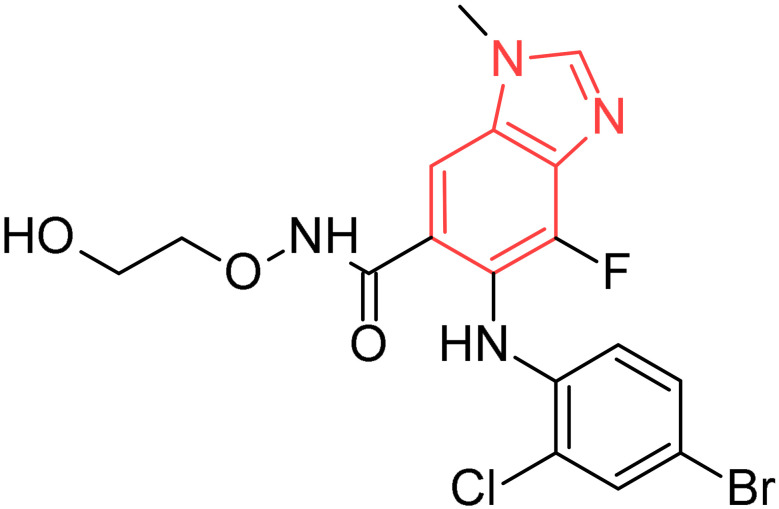	Neurofibromatosis type 1	[36]

Similarly, triazole derivatives represent another important class of heterocycles that have been widely explored for their anticancer and kinase-inhibitory potential. The triazole ring system, a nitrogen-rich five-membered heterocycle including both 1,2,3- and 1,2,4-triazoles, has displayed exceptional promise in cancer therapy due to its stability and strong binding affinity toward kinase domains [37,38]. Triazoles have gained considerable attention because of their broad spectrum of biological activities, including anticancer effects [39,40,41,42]. Several FDA-approved drugs and investigational compounds incorporating triazoles are reported in the literature [43,44,45] (Table 2).

One of the reported synthetic methods to obtain the 1,2,3-triazole ring is initiated by 4-chloroaniline as the starting material, which is diazotised using sodium nitrite in an acidic medium at low temperature, yielding the corresponding aryl diazonium salt. This intermediate then reacts with 2-aminoacetonitrile hydrochloride, yielding 2-(2-(4-chlorophenyl)iminohydrazino)acetonitrile. Upon refluxing in ethanol, this intermediate undergoes intramolecular cyclisation, leading to the formation of the desired 1,2,3-triazole derivative, specifically *N*-(4-chlorophenyl)-2*H*-1,2,3-triazol-4-amine.

In contrast to 1,2,3-triazoles, which are commonly synthesised via azide–alkyne cycloaddition (click chemistry) or diazotisation-based routes, the construction of 1,2,4-triazoles typically follows a different synthetic strategy. One reported method involved a coupling/cyclisation reaction between *N*-arylamidrazones and aldehydes [46], carried out in polyethylene glycol (PEG) as a green solvent and catalysed by ceric ammonium nitrate (CAN, 5%). Heating the reaction mixture under these conditions afforded a series of 3,4,5-trisubstituted 1,2,4-triazoles.

**Table 2:** FDA and investigational anti-cancer compounds containing triazole.

Compound Name	Structure	Target	Reference
Deucravacitinib	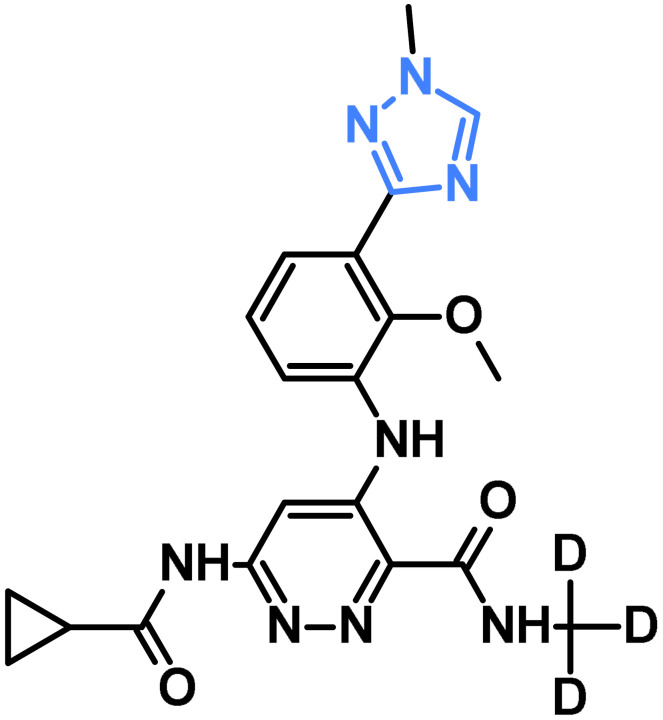	Tyrosine Kinase 2	[43]
Tucatinib	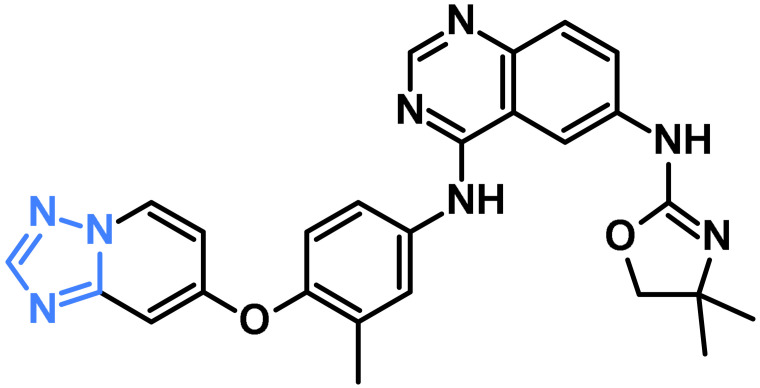	HER2	[44]
Mubritinib	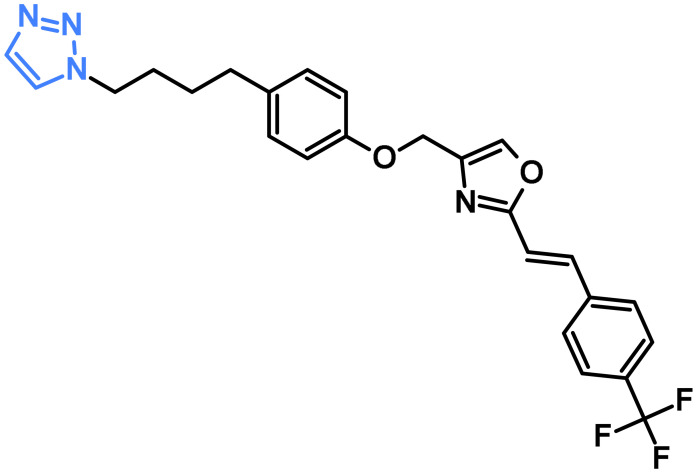	HER2	[45]

### 3.1. Benzimidazole Compounds as Kinase Inhibitors

Over the past decades, benzimidazoles have gained considerable attention for a wide range of biological activities. Among these, their ability to inhibit protein kinases is of particular significance. Since abnormal kinase activity is a hallmark of many cancers, benzimidazole-based compounds have become valuable candidates in the development of anticancer therapeutics targeting these enzymes.

#### 3.1.1. Benzimidazoles Targeting EGFR

**Figure 2:**
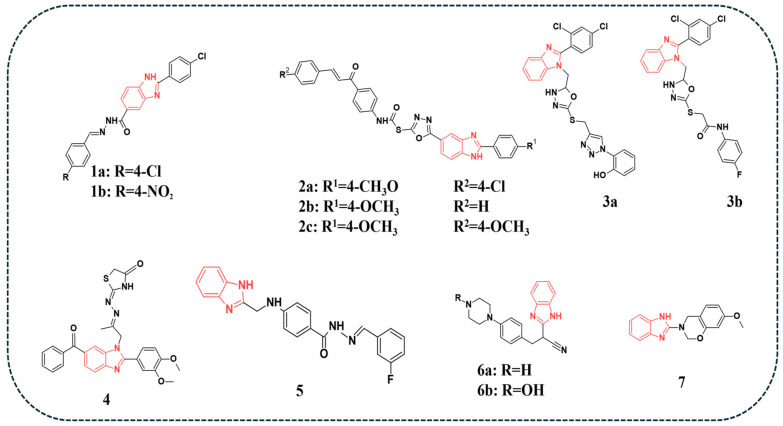
Benzimidazole-containing compounds targeting EGFR, as reported in the literature and discussed in this review; compound codes are assigned in this review for consistency.

Youssif and colleagues synthesised a series of benzimidazole-based derivatives that act as dual inhibitors of EGFR and BRAF. Their antitumour activity was tested against the NCI-60 panel of tumour cell lines. The results showed that compound **1a** (Figure 2) exhibited the most potent inhibitory effect, with IC_50_ values of 0.09 and 0.20 µM against EGFR and BRAF, respectively (Table 3). Molecular docking studies demonstrated that compounds **1a** and **1b** (Figure 2) displayed good binding interactions within the active sites of EGFR and BRAFV600E, supporting their potential as effective inhibitors [47].

A series of benzimidazole–oxadiazole–chalcone hybrids were tested for their inhibitory and antiapoptotic activities against different cell lines [48]. Among the synthesised derivatives, compound **2a** (Figure 2) showed the best IC_50_ values against LOX-IMVI and MCF-7 cell lines (0.80 and 1.10 µM, respectively), while compound **2b** (Figure 2) displayed the highest IC_50_ values of 1.20, 1.30, and 1.40 µM against A-549, Panc-1, and HT-29 cell lines, respectively. Doxorubicin was used as the reference (IC_50_ = 1.21 µM in A-549, 0.90 µM in MCF-7, 1.41 µM in Panc-1, and 1.01 µM in HT-29). The cytotoxicity toward normal MCF-10A cells was also evaluated, where compounds **2a**, **2b**, and **2c** (Figure 2) exhibited cell viability values of 84%, 91%, and 94% at 50 µM, respectively, indicating low toxicity and a favourable selectivity profile. The kinase inhibitory assay showed that compound **2b** exhibited the most potent activity against EGFR, with an IC_50_ value of 0.55 µM, compared to Erlotinib (IC_50_ = 0.08 µM) (Table 3). The derivatives were docked into the active sites of EGFR and BRAF. Among the tested compounds, **2a, 2b,** and **2c** exhibited the most favourable binding scores in both active sites. Additionally, in silico ADMET evaluations were performed, revealing that these compounds (i.e., **2a, 2b**, and **2c**) possessed relatively comparable pharmacokinetic profiles to Erlotinib [48].

A novel series of benzimidazoles was reported and tested for their activity against MDA-MB-231, SKOV3, and A549 cell lines, and the in vitro EGFR inhibitory effect was also evaluated [49]. Among the tested compounds, compounds **3a** and **3b** (Figure 2) demonstrated the most promising activity, evidenced by the lowest IC_50_ values in each cell line. Moreover, the EGFR kinase assay showed that compounds **3a** and **3b** exhibited the best inhibitory effects with IC_50_ values of 0.33 and 0.38 μM, respectively, as compared with the reference drug Erlotinib (IC_50_ = 0.39 μM) (Table 3). Docking studies revealed that the presence of key hydrogen-bonding interactions, as observed in compounds **3a** and **3b**, accounted for their superior binding affinities and enhanced potency. Furthermore, in silico toxicity predictions suggested that compounds **3a** and **3b** exhibited lower toxicity profiles compared to Erlotinib [49].

El-Meguid and colleagues have reported the synthesis of novel benzimidazole derivatives that were tested against HeLa cell lines and further evaluated for their multi-kinase inhibitory activity, including EGFR. Several tested compounds showed promising IC_50_ values. Compound **4** (Figure 2) demonstrated superior activity against the HeLa cell line, exhibiting an IC_50_ value of 1.44 µM, compared to Doxorubicin (IC_50_ = 2.05 µM). The compound also showed a favourable safety profile toward normal WI-38 cells (IC_50_ = 77.34 µM), comparable to Doxorubicin (IC_50_ = 79.7 µM). Furthermore, compound 4 exhibited potent inhibition of EGFR with an IC_50_ of 0.109 µM, compared to Erlotinib (IC_50_ = 0.079 µM) and Nazartinib (IC_50_ = 0.160 µM) (Table 3). Cell cycle analysis further revealed that compound **4** demonstrated a substantial increase in the proportion of cells, elevating the pre-G1 population by 27-fold and the G2/M phase population by 10-fold, indicating strong apoptotic activity and cell-cycle arrest [50].

In a related investigation, Mirgany and colleagues synthesised a series of novel benzimidazole hybrids and evaluated their inhibitory activity against EGFR and other kinases. Among the tested derivatives, compound 5 (Figure 2) exhibited notable cytotoxic activity with IC_50_ values of 13.44, 9.39, and 11.64 µM against HCT-116, HepG2, and MCF-7 cell lines, respectively, while showing a significantly higher IC_50_ value of 56.46 µM against WI-38 normal cells, indicating selectivity toward cancer cells. In comparison, Doxorubicin showed IC_50_ values of 5.23, 4.50, and 1.61 µM against HCT-116, HepG2, and MCF-7 cell lines, respectively, and 6.72 µM against WI-38 cells. In vitro protein kinase inhibition test demonstrated that compound **5** displayed the best IC_50_ value of 30.1 nM against EGFR (Table 3), along with notable inhibition of other kinases. Cell cycle analysis revealed that compound **5** induced a significant cell cycle arrest at the G1 phase in HepG2 cancer cells, highlighting its anticancer potential. Docking results demonstrated that compound **5** exhibited more extensive and specific interactions with EGFR, suggesting a higher binding affinity and specificity, correlating well with its biological activity and G1 phase cell cycle arrest [51].

In a study by Sarita and co-authors, benzimidazole analogues were synthesised, and a sulforhodamine B (SRB) assay was carried out to assess their anticancer activity against the lung carcinoma cell line, A549. Compounds **6a** and 6b (Figure 2) demonstrated the highest cytotoxic activity against the tested cell line with CTC_50_ (Cytotoxic Concentration 50%) values of 117 and 114 μg/mL, respectively. The authors concluded that compound **6b** exhibited the most favourable binding interactions in the molecular docking studies, suggesting its potential as a promising anticancer candidate for further investigation [52].

Moreover, Gali and colleagues reported novel oxazine analogues bearing benzimidazole and tested them against two breast cancer cell lines, MCF-7 and MDA-MB-231, along with the control, Doxorubicin. Compound **7** (Figure 2) demonstrated the highest IC_50_ values in both cell lines (8.60 and 6.30 μM, respectively). The safety of the compounds was assessed using MCF-10A normal cells, where no adverse morphological changes were observed. Additionally, compound **7** scored the highest binding affinity against the docked active site of EGFR (PDB ID:2J6M). This superiority was attributed to a key interaction with Met793, exhibiting a bond distance of 2.94 Å in the cavity of EGFR. Pharmacokinetic analysis confirmed that all derivatives complied with standard drug-likeness criteria, indicating overall favourable absorption properties and bioavailability [53].

**Table 3:** EGFR inhibitory activity (IC_50_) of benzimidazole-based compounds compared with the reference drug Erlotinib. Values are expressed in μM or nM.

Compound No.	EGFR Inhibitory Activity		Reference
	(IC_50_)	Reference Drug (Erlotinib)	
**1a**	0.09 µM	0.08 µM	[47]
**1b**	0.11 µM		
**2a**	0.80 µM	0.08 µM	[48]
**2b**	0.55 µM		
**2c**	0.90 µM		
**3a**	0.33 µM	0.39 µM	[49]
**3b**	0.38 µM		
**4**	0.109 µM	0.079 µM	[50]
**5**	30.1 nM	61.1 nM	[51]

#### 3.1.2. Benzimidazoles Targeting PI3K/Akt/mTOR Pathway

**Figure 3:**
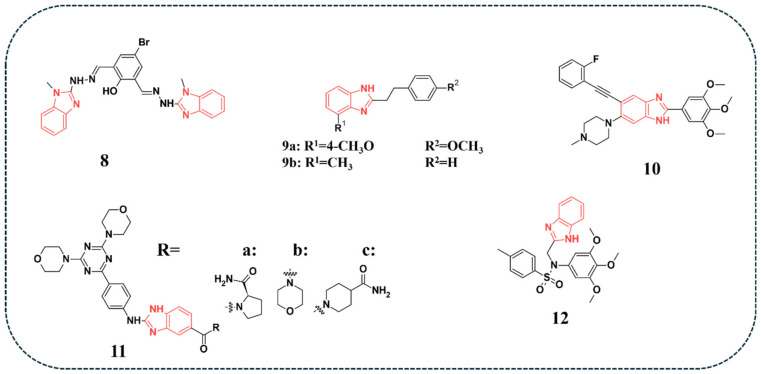
Benzimidazole-containing compounds targeting PI3k/Akt/mTOR pathway, as reported in the literature and discussed in this review; compound codes are assigned in this review for consistency.

PI3k and other kinases are known to be involved in multiple myeloma, a malignancy characterised by the production of abnormal antibodies by abnormal plasma cells, by regulating cell survival, growth, and resistance to treatment. Xu and colleagues synthesised a novel compound, **8** (Figure 3), and aimed to test its activity against multiple myeloma. Inhibitory tests were performed against U266 and RPMI 8226 cell lines at different times and concentrations. The tested compound displayed IC_50_ values of 4.3 μM against U266 and 5.1 μM against RPMI8226. The compound exhibited a time-dependent decrease in cell viability, with treatment at 5 μM over 12, 24, and 48 h resulting in a progressive reduction in viable cells in comparison with the untreated control. The authors stated that the compound-induced cell apoptosis in both cell lines was attributed to inhibiting couple signalling pathways, including the PI3K/Akt/mTOR signalling pathway, as evidenced by the PI3K, Akt, and mTOR protein expression. Moreover, Western blot assays demonstrated that compound **8** exerted its anti-multiple myeloma by inhibiting NF-κB and PI3K/AKT/mTOR signalling, causing cell cycle arrest. Toxicity against normal cells was assessed by incubating the compound with PMBCs and U266 cells for 12 h. Staining results revealed significant elimination of U266 cells and insignificant toxicity to the PMBCs [54].

Other researchers reported the synthesis of a series of novel benzimidazole derivatives and investigated their effects on the mTOR pathway. Tests were done on MCF-7 using a colourimetric cell-based enzyme-linked immunosorbent assay (ELISA). MTT assay was performed at 48 and 72 h, and the compounds **9a** and **9b** (Figure 3) showed the best IC_50_ values, 5.52 and 4.62 µM, respectively, at the end of 72 h. To assess mTOR inhibition, the Ser2488 phosphorylation site on the mTOR was found to be significantly reduced, providing evidence for its inhibition [55].

Additional studies by Li and co-authors reported the synthesis of novel benzimidazole derivatives and tested their metabolic activity against HeLa, SiHa, and Ca Ski, and evaluated their toxicity on normal cells of liver (LO2) and kidney (HEK-293T). The results revealed that most of the compounds exhibited strong activity against the tested cell lines, but were associated with significant toxicity, except for compound **10** (Figure 3). Compound **10** showed an IC_50_ value of 3.38 μM, while displaying minimal toxicity toward LO2 and HEK-293T cell lines, with IC_50_ values of 21.08 and 23.96 μM. The toxicity of compound **10** was also evaluated in zebrafish embryos and displayed a good safety profile at standard concentrations. The authors proved that compound **10** suppressed the PI3K/Akt/mTOR pathways, which in turn prevented SiHa cells from growing. Molecular docking studies were done on PI3Kα enzyme (PDB ID: 8EXU). Authors also stated that the key interactions found were a hydrogen bond with GLN993, HIS994 of PI3Kα [56].

The synthesis and evaluation of the inhibitory activity of triazines bearing benzimidazole against various cancer cell lines was reported by Wu and co-authors [57]. Compounds **11a** and **11b** (Figure 3) were further evaluated for their inhibitory effects against different isoforms of class 1 PI3K. Of the tested compounds, **11a** exhibited comparable inhibitory effects when tested against PI3K alpha and beta isoforms. However, a significant reduction in activity was observed against the gamma isoform. Several derivatives, including **11a** and **11b** with acetamide substituents linked to the carboxyl group of the benzimidazole structure, demonstrated significant inhibitory activity against PI3Kd. Compound **11c** exhibited exceptional activity with an IC_50_ value of 2.3 nM for PI3Kd (Table 4). Western blot assay revealed that compounds **11a** and **11b** resulted in almost a complete inhibition of the phosphorylation of Akt and p70S6K, indicating strong suppression of the PI3K/AKT/mTOR signalling pathway. Docking studies for the two compounds, **11a** and **11b**, revealed a key interaction between the oxygen atom of the morpholine ring in the two compounds and the conserved valine residues within the active sites of PI3Kα, PI3Kδ, and mTOR, highlighting the importance of this moiety for target binding and inhibitory activity [57].

Novel tertiary sulphonamide derivatives containing benzimidazole were synthesised and evaluated for their activity against MGC-803, PC-3, and MCF-7 cell lines [58]. Of the tested derivatives, compound **12** (Figure 3) demonstrated superior activity, achieving the highest IC_50_ values of 1.02, 3.34, and 5.40 μM against MGC-803, PC-3, and MCF-7, respectively. Western blot analysis revealed that compound **12** markedly reduced the expression of phosphorylated Akt and c-Raf, indicating that it exerts its anti-cancer effects in gastric cancer cell lines by disrupting both AKT/mTOR and RAS/Raf/MEK/ERK signalling pathways [58].

**Table 4:** PI3K/mTOR inhibitory activity (IC_50_) of benzimidazole-based compounds compared with the reference drug gedatolisib. Values are expressed in nM.

Compound No.	Inhibitory Activity					Reference
	(IC_50_)					
**11**	PI3Ks				mTOR	[57]
	PI3Ka	PI3Kb	PI3Kγ	PI3Kδ		
**a**	7.3 nM	21.3 nM	444 nM	5.1 nM	5.6 nM	
**b**	20.1 nM	28 nM	>1000 nM	13 nM	12.9 nM	
**c**	14.6 nM	34 nM	849 nM	2.3 nM	15.4 nM	
Reference drug (gedatolisib)	6 nM	-	-	-	2.1 nM	

#### 3.1.3. Benzimidazoles as CDK Inhibitors

**Figure 4:**
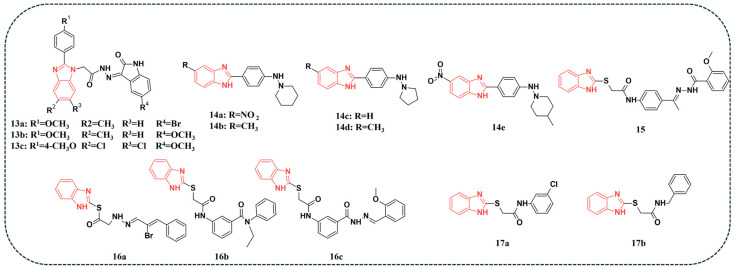
Benzimidazole-containing compounds targeting CDK, as reported in the literature and discussed in this review; compound codes are assigned in this review for consistency.

A series of benzimidazole–oxindole hybrids were synthesised and evaluated for their antitumor activity against several human cancer cell lines, NCI-60 panel, PANC-1, and MG-63 [59]. Screening against the NCI-60 panel revealed that several compounds exhibited pronounced antiproliferative activity. Compound **13a** (Figure 4) showed the highest IC_50_ value of 1.88 µM against PANC-1, while compound **13b** (Figure 4) demonstrated the best antiproliferative activity against MG-63 with an IC_50_ value of 0.99 µM. Among the series, compound **13c** emerged as the most potent dual inhibitor, displaying IC_50_ values of 0.04 µM for CDK2 (Table 5) and 0.021 µM for GSK-3β, with marked selectivity over other kinases. This dual activity might be attributed to the comfortable fitting of compound **13c** (Figure 4) within the catalytic sites of both kinases, adopting a comparable binding pose as demonstrated by docking studies. In this orientation, the oxindole unit anchors in the hinge region, where its CONH group forms hydrogen bonds with Glu81 and Leu83 in CDK2, and with Asp133 and Val135 in GSK-3β. Meanwhile, the phenyl ring of the oxindole core is held in place through hydrophobic contacts with nearby amino acid side chains, further stabilising the complex [59].

In another study, several 2-phenylbenzimidazole hybrids were designed and synthesised, and their activity against 60 NCI-cancer cell lines was evaluated. Compounds **14a-e** (Figure 4) were selected as they displayed the highest percentages of growth inhibition in the tested cell lines. An enzyme assay was performed with the selected compounds, and the results revealed that compounds **14a** and **14e** exhibited the strongest IC_50_ values against CDK6 and Aurora A kinase. For CDK6, compounds **14a** and **14e** showed IC_50_ values of 0.197 and 0.172 μM, respectively (Table 5). Regarding Aurora A kinase, compounds **14a** and **14e** achieved IC_50_ values of 0.074 and 0.062 μM, respectively. Compounds **14a** and **14e**, the most potent Aurora A kinase inhibitors, formed hydrogen bonds with Ala213 in addition to hydrophobic interactions, aided by the nitro group on their benzimidazole ring that enabled the benzimidazole nitrogen to act as an H-bond donor. In contrast, compounds **14b** and **14d** failed to form hydrogen bonds, suggesting a weaker affinity. The majority of the selected compounds showed favourable binding free energies with CDK6. However, their affinities were somewhat lower than those observed with Aurora A kinase, likely due to CDK6’s reliance on hydrophobic contacts and fewer hydrogen bonds. The co-crystal ligand interacted with Glu99 and Ile19, and the experimental results were consistent with the docking predictions [60].

A novel series of benzimidazole derivatives was synthesised, and their antiproliferative activity was evaluated against A549 and PC-3 cell lines [61]. Several derivatives displayed stronger activity than the reference drugs, 5-fluorouracil and gefitinib. Notably, compound **15** (Figure 4) achieved the highest potency against A549 cells, with 66.10% inhibition at 10 μM and an IC_50_ value of 7.19 μM, outperforming the activity of 5-fluorouracil (10.56 μM) and gefitinib (27.07 μM). Mechanistic assays demonstrated that compound **15** induced apoptosis, increased ROS levels, caused mitochondrial membrane potential collapse, and arrested the cell cycle at the G2/M phase. Western blot analysis revealed that compound **15** significantly decreased the levels of cell cycle proteins, including CDK-1 [61].

Tahlan and colleagues highlighted the potential of heterocyclic benzimidazole scaffolds as potential anticancer candidates through in vitro and in silico approaches. A panel of benzimidazole derivatives was screened against HCT116 to test their anticancer activity. Compound 16a (Figure 4) exhibited the best IC_50_ value of 0.46 µM. Docking studies were performed on CDK-8 (PDB code: 5-FGK) and ER-α (PDB code: 3ERT). Several compounds, including 16a, 16b, and 16c (Figure 4), demonstrated favourable docking scores and consistent in vitro activity. For CDK8, frequent contacts were observed with Lys52, Ala100, Ala155, Asp173, Tyr32, and Val27, while for ER-α, important interactions involved Thr347, Asp351, Val534, Leu346, and Cys530. Among them, **16b** (Figure 4) showed the strongest docking score against CDK8 (−9.686), whereas compound **16c** achieved the best binding to ER-α (−8.986) [62].

A novel series of benzimidazole derivatives was evaluated for antiproliferative activity against the NCI-60 human cancer cell line panel. Among the synthesised compounds, **17a** and **17b** (Figure 4) exhibited the most notable activity, selectively inhibiting the growth of HCT-116 colon cancer and TK-10 renal cancer cell lines, respectively. Consequently, these two derivatives were further investigated through molecular docking using the crystal structure of CDK2 (PDB ID: 5ANJ). The docking analysis revealed that both compounds bound favourably within the ATP-binding pocket of CDK2. Compound **17a** achieved a docking score of −4.36 kcal/mol through a stabilising π–H interaction with Ile10. In comparison, compound **17b** demonstrated a stronger binding affinity with a score of −6.34 kcal/mol, even surpassing the reference inhibitor ZXC (−5.35 kcal/mol) by engaging in multiple stabilising interactions, including hydrogen bonds with Leu83 and Lys33. Despite these promising docking results, the in vitro CDK2 kinase inhibition and cytotoxicity assays confirmed that **17a** and **17b** were significantly less potent than the reference drug, Imatinib (Table 5), highlighting the need for further structural optimisation and mechanistic studies to enhance their anticancer efficacy [63].

**Table 5:** CDK inhibitory activity (IC_50_) of benzimidazole-based compounds compared with a reference drug. Values are expressed in μM or nM.

Compound No.	CDK Inhibitory Activity		Reference
	(IC_50_)	Reference Drug	
	CDK2		[59]
**13a**	0.40 μM	Staurosporine 0.022 µM	
**13b**	0.09 μM		
**13c**	0.04 µM		
	CDK6		[60]
**14a**	0.197 µM	Staurosporine 0.319 µM	
**14b**	1.49 µM		
**14c**	-		
**14d**	0.761 µM		
**14e**	0.172 µM		
	CDK2		
**17a**	423.81 nM	Imatinib 155.09 nM	[63]
**17b**	330.21 nM		

### 3.2. Triazole Compounds as Kinase Inhibitors

Triazole-based compounds have attained considerable interest in anticancer drug discovery owing to their ability to modulate key molecular targets such as protein kinases [64,65,66]. The triazole ring, with its electron-rich and hydrogen-bonding capabilities, can serve as a bio-isostere for amide or heteroaromatic groups, facilitating strong interactions within the ATP-binding pocket of kinases. As a result, numerous triazole derivatives have been developed as inhibitors of clinically relevant kinases, including EGFR [67], VEGFR-2 [68], CDK2 [65], PI3K [69], and mTOR [70].

The following section outlines key advancements in triazole-based anticancer research, summarising representative studies that underscore their therapeutic potential.

#### 3.2.1. Triazoles Targeting EGFR

**Figure 5:**
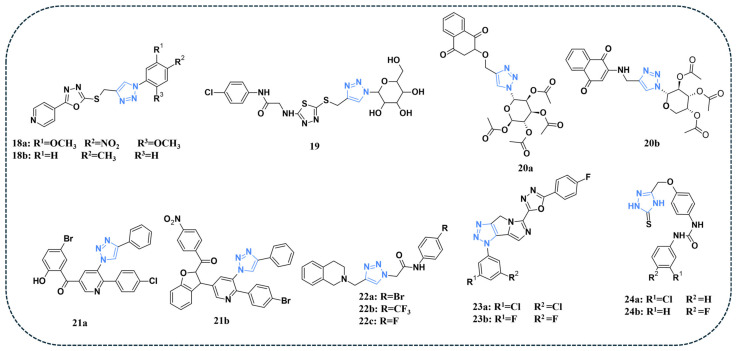
Triazole-containing compounds targeting EGFR, as reported in the literature and discussed in this review; compound codes are assigned in this review for consistency.

A series of novel compounds incorporating 1,2,3-triazole were synthesised and evaluated for their activity against HCT-116, HePG-2, and MCF-7 cancer cell lines [71]. Compound **18a** (Figure 5) demonstrated the best activity against HCT-116 and HePG-2 cell lines with IC_50_ values of 9.58 and 7.83 µM, respectively. For the MCF-7 cell line, compound **18b** (Figure 5) demonstrated the highest activity, with an IC_50_ value of 5.56 µM. The EGFR enzymatic assay confirmed the potent inhibitory effect of compound **18a** on EGFR (IC_50_ = 0.313 µM) (Table 6) and promoted G0/G1 cell-cycle arrest via apoptosis in HepG-2 cells [71].

A study by Hussein et al. reported the synthesis of a series of novel thiadiazole–triazole hybrid glycosides. The compounds were evaluated against human HCT-116, HepG-2, and MCF-7 cancer cell lines [72]. Compound **19** (Figure 5) displayed superior antiproliferative activity with an inhibition percentage greater than 80%. Additionally, compound **19** also showed the best cytotoxic effects against the tested cell lines. Moreover, compound **19** exhibited the most potent dual inhibitory activity, with IC_50_ values of 0.21 µM against EGFR (Table 6) and 0.62 µM against VEGFR-2. Docking simulations demonstrated that compound **19** exhibited strong affinity toward EGFR (PDB ID: 1M17), with stable binding interactions involving hydrogen bonds between its sugar hydroxyl groups and the residues Gln767 and Met769, as well as an additional hydrogen bond formed via its amide NH group with Asp831. In the VEGFR-2 active site (PDB ID: 4ASD), compound **19** showed even greater predicted binding energy, forming key hydrogen bonds with Asp1046 and Lys868. Its glycosidic portion was also engaged in further stabilising interactions with Glu917 and Cys919, contributing to its enhanced fitting within the binding pocket [72].

A series of novel triazoles comprising 1,4-naphthoquinone hybrids was reported and assessed for their anticancer activity against the A549 cell line [73]. Two distinct series were designed: one incorporating an oxygen atom as a linker between the triazole and the 1,4-naphthoquinone moiety, and the other containing an NH group serving as the connecting linker. Among the tested compounds, compounds **20a** and **20b** (Figure 5) displayed the highest anticancer activity with IC_50_ values of 5.17 and 7.89 μM, respectively. Docking studies against EGFR (PDB ID: 1M17) were performed, and compound 20a achieved the most favourable docking score (−6.78 kcal/mol), stabilised through several hydrogen bonds with Lys692, Lys704, and Met769, in addition to π-alkyl interactions with Val702, Ala719, and Leu768. In contrast, compound **20b** demonstrated a moderate docking score (−5.65 kcal/mol), engaged mainly in hydrogen bonding with Met769 and supported by π-alkyl contacts involving Ala719, Val702, and Leu820 [73].

Elumalai and co-authors reported the synthesis of novel triazolylpyridine and triazolylpyridinylbenzofuran hybrids and tested their therapeutic potential against the EGFR via in silico studies. Compound **21a** (Figure 5) from the triazolyl-pyridine derivatives and compound **21b** (Figure 5) from the benzofuranyl-triazolyl-pyridine set exhibited stronger binding affinities (−11.81 and −9.17 kcal/mol, respectively) than the reference drug, gefitinib. Compound **21a** exhibited the most favourable interaction pattern with EGFR, positioning itself firmly within the active pocket through a network of hydrogen bonds along with numerous hydrophobic and π-based contacts, which collectively contributed to its exceptional binding affinity. In comparison, compound **21b** also demonstrated robust stabilisation within the binding site, largely driven by π–π-cation and π–π-anion interactions, resulting in a docking profile that surpassed the reference drug gefitinib [74].

Chabhadiya and colleagues reported in vitro and in silico assessment of tetrahydroisoquinoline derivatives against the A549 lung cancer cell line. Compounds **22a**, **22b**, and **22c** (Figure 5) demonstrated the lowest IC_50_ values of 28.38, 14.46, and 22.50 μg/mL, respectively, indicating the superior inhibitory activity of compound **22b** against the tested cell line. Docking studies were performed on the EGFR protein in complex with MTX-531 (PDB ID: 8SC7). Compound **22b** demonstrated the best docking score, having a value of −8.5 Kcal/mol. Molecular dynamics simulations further validated that the complexes of compounds **22b** and **22c** with EGFR remained stable throughout the 200 ns simulation period [75].

A novel series of 1,3,4-oxadiazole incorporating triazole hybrids was synthesised, and their activity was assessed against A549 and NCI-H4607 cell lines [76]. Compounds **23a** and **23b** (Figure 5) demonstrated the activity against the tested cell lines with IC_50_ values of 3.46 and 5.43 μM, respectively. Furthermore, evaluation of the EGFR inhibitory activity indicated that compounds **23a** and **23b** exhibited remarkable potency against EGFR, with IC_50_ values of 0.29 and 0.37 µM, respectively (Table 6), making them the most effective inhibitors within the series. Docking studies were carried out on the EGFR TKD protein, and compound **23b** displayed the highest binding energy of −7.4 Kcal/mol, mimicking the binding pattern of Erlotinib. Its stability was ensured through hydrogen bonds with Met769 and Lys721, a halogen bond with Pro770, and a π–σ interaction with Val702. Additional hydrophobic and van der Waals contacts involving residues such as Ala719, Leu820, Leu694, Leu768, and Phe832 further reinforced the complex, highlighting the compound’s potent inhibitory potential [76].

**Table 6:** EGFR inhibitory activity (IC_50_) of triazole-based compounds compared with a reference drug. Values are expressed in μM or nM.

Compound No.	EGFR Inhibitory Activity		Reference
	(IC_50_)	Reference Drug	
**18a**	0.313 µM	Erlotinib 0.039 µM	[71]
**19**	0.21 µM	Erlotinib 0.18 µM	[72]
**23a**	0.37 µM	Erlotinib 0.44 µM	[76]
**23b**	0.29 µM		
**24a**	630 µM	Gefitinib 0.0094 µM	[77]
**24b**	0.0351 µM		

Türe and co-authors reported a novel series of triazole–urea hybrids and evaluated the antiproliferative activity of the synthesised compounds on the MCF7 breast cancer cell line and the L929 fibroblast cell line [77]. Among the tested compounds, **24a** (Figure 5) demonstrated the highest activity against MCF7 with an IC_50_ value of 56.97 µM, and low toxicity against L929 (IC_50_ = 1651 µM). Compound **24b** (Figure 5) showed superior inhibition of EGFR with an IC_50_ value of 0.0351 µM, while **24a** displayed an IC_50_ value of 630 µM (Table 6). Docking studies were performed utilising the crystal structure of the EGFR protein in complex with the inhibitor TAK-285 (PDB ID: 3POZ). Compound **24b** displayed a distinct binding mode, forming a water-mediated hydrogen bond with Leu718, a direct hydrogen bond with the gatekeeper Thr790, and additional interactions with Cys775 and Asp855. Its phenyl ring was also engaged in π–π stacking with Phe997, suggesting stronger stabilisation within the EGFR ATP-binding pocket and contributing to its potent inhibitory profile [77].

#### 3.2.2. Triazoles Targeting PI3K/Akt/mTOR Pathway

**Figure 6:**
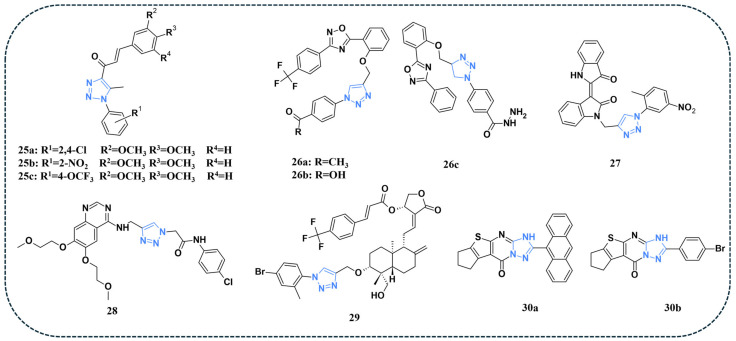
Triazole-containing compounds targeting PI3K/Akt/mTOR, as reported in the literature and discussed in this review; compound codes are assigned in this review for consistency.

Shaheen and co-authors synthesised and assessed the antiproliferative properties of a series of 1,2,3 triazole hybrids against the NCI 60 panel. Of all evaluated compounds, **25a**, **25b**, and **25c** (Figure 6) displayed the highest growth inhibition on the panel of cell lines, with values greater than 80%. The EGFR inhibition assay revealed that compound **25b** demonstrated a superior IC_50_ value of 0.155 μg/mL. Gene expression of mTOR was assessed, and results indicated that treatment of HCT-116 cells with compound **25b** led to a three-fold reduction in mTOR expression [78].

Furthermore, Ayoup and colleagues synthesised a series of 1,2,4-oxadiazole-linked 1,2,3-triazole hybrids and evaluated their anticancer properties against A549 and Caco-2 cancer cell lines, as well as the human lung fibroblast (WI38) [79]. An MTT assay revealed that compounds **26a, 26b,** and **26c** (Figure 6) exhibited the highest activity against the tested cancer cell lines. Further evaluations were therefore conducted for compounds **26a**, **26b**, and **26c** to investigate their effects on the PI3K, mTOR, EGFR, and p53 pathways in both cancer models. In A549 cells, compound **26c** displayed the strongest suppression of PI3K, mTOR, and EGFR, accompanied by robust p53 upregulation, indicating a strong ability to modulate the EGFR/PI3K/mTOR signalling cascade. Compound **26b** demonstrated balanced downregulation across PI3K, mTOR, and EGFR with significant p53 activation, while compound **26a** showed slightly weaker, yet still notable, modulation of these pathways. In the Caco-2 cell line, compound **26b** exhibited the strongest suppression of PI3K, mTOR, and EGFR, together with the highest induction of p53, suggesting effective regulation of the oncogenic signalling network. Compound **26c** also demonstrated considerable inhibition of these kinases with substantial p53 activation, whereas compound **26a** showed comparatively weaker, though still significant, effects. Docking studies were performed using the EGFR–gefitinib complex X-ray structure (PDB: 2ITY). The investigated compounds **26a**, **26b**, and **26c** displayed stronger binding affinities toward the EGFR active site than gefitinib, supporting their proposed mechanism of action. The docking analysis revealed key interactions, including hydrogen bonding with Met793, hydrophobic stabilisation with residues Leu718, Val726, Ala743, Lys745, and Leu844, and occupation of hydrophobic pockets I and II by the aryl substituents [79].

A novel series of indurubin triazole hybrids was synthesised, and their activity against HCCLM3 and Hep3B was evaluated [80]. Among the synthesised compounds, compound **27** (Figure 6) demonstrated the strongest cytotoxic activity against the HCCLM3 cell line. Western blot analysis was performed to investigate the HGF-driven c-MET activation in hepatocellular carcinoma. The results demonstrated that the phosphorylation of c-MET was reduced after treatment with compound **27** along with HGF, indicating that compound **27** prevented c-MET activation, and hence suppressed the activation of the downstream PI3K/Akt/mTOR signalling pathway [80].

A novel quinazoline-containing 1,2,3-triazole compound was synthesised, and its activity was assessed against different cell lines (A549, MCF7, K562, and HFF2) [81]. An MTT assay showed that the compound **28** (Figure 6) exhibited a two-fold increase in effect than the reference drug Erlotinib against the tested cell lines. The compound demonstrated a dose- and time-dependent reduction in cell viability, with IC_50_ values of 35.70, 19.50, and 5.95 μM for A549, MCF7, and K562 cells, respectively, after 72 h of treatment. However, it was noted that compound **28** displayed some toxicity in the case of HFF2 cells, though to a lesser extent compared to cancer cells. Compound **28** significantly downregulated key survival genes, most notably EGFR, VEGFR2, and mTOR in A549, MCF7, and K562 cells, thereby promoting mitochondrial-mediated apoptotic cell death [81].

Vishwanadham et al. synthesised a series of novel andrographolide analogues containing triazole and evaluated the cytotoxic effects of the compounds against several breast cancer cell lines (SKBR-3, MCF-7, MDAMB-231, T-47D, and BT-474). Among the tested analogues, compound 29 (Figure 6) demonstrated superior efficacy against almost all cell lines. Mechanistic studies revealed that the compound effectively inhibited the PI3K/AKT/mTOR signalling pathway, even in oestrogen-stimulated cells, and showed partial synergy with tamoxifen. Moreover, kinase assays confirmed strong inhibitory activity of compound **29** against PI3Kα. Docking studies were performed in the PI3Kα active site (PDB ID: 3TL5), and the results indicated that compound **29** fits well in the active site, with a docking score of −9.87 and ΔG = −985.2 kcal/mol. The stability of the complex was largely attributed to two critical hydrogen bonds: the methoxy oxygen on the decahydronaphthalene ring interacted with Lys890 in the hinge region, and the hydroxyl group at the 5th position formed a hydrogen bond with Asp950, effectively anchoring the ligand [82].

A study by Elsenbawy and co-authors reported a novel series of pyridine derivatives incorporating 1,2,4 triazole and evaluated their potency against MCF-7, HCT-116, and PC-3 cancer cell lines. Among the tested derivatives, compound **30a** (Figure 6) displayed the highest inhibitory percentages and superior IC_50_ values of 14.5, 57.01, and 25.23 µM, respectively. However, against the Bj-1 cell line, derivative **30b** (Figure 6) exhibited a better safety profile than **30a**. Docking studies were performed on EGFR and PI3K active sites (PDB codes: 1M17 and 3L54, respectively). Compound **30b** exhibited favourable EGFR binding via key interactions with Met769, Val702, and Glu738. In contrast, the bulkier derivative **30a** lost Met769 contact but demonstrated an improved fit within the PI3K binding site through H-bonds with Asp836 and Asp964, resulting in a selective shift toward PI3K [83].

#### 3.2.3. Triazoles as CDK Inhibitors

**Figure 7:**
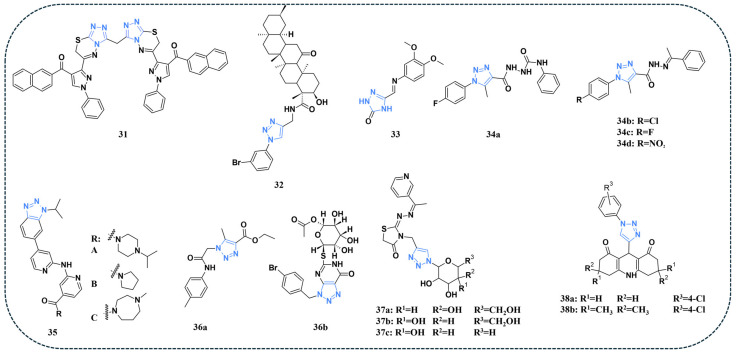
Triazole-containing compounds targeting CDK, as reported in the literature and discussed in this review; compound codes are assigned in this review for consistency.

Salem and co-authors synthesised a novel series of 6-pyrazolyltriazolothiadiazine derivatives and assessed their activity against the MCF-7 breast cancer cell line and the normal breast cell line MCF-10A. The bis-heterocyclic compound **31** (Figure 7) displayed a significant cytotoxic activity against the MCF-7 cell line, with an IC_50_ value of 0.39 μM. The compound also exhibited a considerable safety profile against MCF-10A, with an IC_50_ value exceeding 50 μM. Furthermore, the kinase inhibition activity of compound **31** was evaluated. An IC_50_ value of 19.6 nM was displayed in the case of EGFR, while the reference Erlotinib demonstrated an IC_50_ value of 67.3 nM. For CDK-2, compound **31** demonstrated an IC_50_ value of 87.9 nM, while the reference drug roscovitine displayed an IC_50_ value of 140 nM (Table 7) [84].

Rehman and co-authors reported the semisynthetic development of novel alicyclic triterpene–triazole derivatives from Boswellia sacra gum resin. The synthesised compounds were tested against MCF-7, MDA-MB-231, and MCF-10A. Compound **32** (Figure 7) displayed the best activity against the tested cell lines. Docking simulations were performed on the CDK enzyme (PDB ID: 7SJ3), and compound **32** revealed the highest activity, achieving a docking score of −7.70 kcal/mol and forming hydrogen bonds with Glu94 (2.04 Å) and Val96 (2.22 Å). Moreover, hydrophobic interactions were observed with Phe93, Ile12, and Gln98, stabilising the triazole and bromophenyl rings [85].

Balavanthapu and Vedula synthesised a novel series of l 5-((Phenylimino)methyl)-1,2,4-triazol-3-one derivatives and evaluated the antiproliferative activities of the compounds against HCT-116, A-549, and PANC-1 cell lines. Among the derivatives, the MTT assay revealed that compound **33** (Figure 7) displayed the highest IC_50_ values against all tested cell lines. Docking simulations were performed on EGFR (PDB ID: 6LUD) and CDK-4 (PDB ID: 7SJ3) and demonstrated binding energies ranging from −5.2 to −6.8 kcal/mol for EGFR and −5.6 to −7.2 kcal/mol for CDK, indicating that most of the compounds demonstrated strong affinities [86].

A study by Al-Karmalawy and his colleagues reported the synthesis of 1,2,3-triazole derivatives, and the compounds were screened against a panel of cancer cell lines, human skin fibroblast cell line (HSF), and the normal oral epithelial cell line (OEC) [87]. Among the tested compounds, **34a** (Figure 7) displayed the best average growth inhibition (GI%) when compared to the reference drug doxorubicin and the other derivatives. Despite the potency of compound **34a**, compounds **34b**, **34c**, and **34d** (Figure 7) were prioritised as leads since they showed stronger VEGFR-2 and telomerase downregulation. Apoptosis-related markers were also investigated, and compound **34b** was found to significantly reduce the levels of CDK (2,4, and 6). Moreover, caspase activity was nearly doubled compared to the control. Docking studies were performed on VEGFR-2 (PDB ID: 4ASD) and telomerase (PDB ID: 5CQG) active sites. The results confirmed that compounds **34b**, **34c**, and **34d** bound VEGFR-2 and telomerase at key residues with affinities comparable to reference inhibitors [87].

Zhong and colleagues synthesised a panel of novel derivatives based on AZD5438 [88], a pan-CDK inhibitor originally reported by AstraZeneca that exhibits low-nanomolar inhibitory activity toward CDK9. Among the tested compounds, **35a**, **35b**, and 35c (Figure 7) displayed significant activity against CDK9 with an IC_50_ value of 3.37, 1.32, and 2.90 nM, respectively. Moreover, these three compounds showed high potency against HCT116 and HT-29 cell lines. Additionally, compound **35c** demonstrated superior selectivity, exerting stronger inhibitory activity on CDK9 (Table 7) while sparing other CDK isoforms (CDK1, 2, 4–8, and 12), thereby highlighting its potential as a more specific CDK9 inhibitor [88].

A series of novel compounds was synthesised, and their activity was tested against MCF-7, HEP-2, and HCT-116 cell lines. Among the synthesised compounds, compound **36a** (Figure 7) exhibited the best inhibition percentage against HEP2 with a value of 84.97%. Moreover, compound **36b** (Figure 7) displayed the highest inhibition rate against HCT and MCF-7, with inhibition percentages of 90.37% and 63.91%, respectively. Compounds **36a** and **36b** exhibited a favourable safety profile. Docking simulations were performed on the CDK-2 active site (PDB: 2A4L), and the two compounds (**36a** and **36b**) displayed good binding to the active site, with compound **36b** achieving the highest docking energy score [89].

Another study reported the synthesis of novel glycoside-based hybrids as potential multi-target kinase inhibitors [65]. The synthesised compounds were screened against HepG-2 and MCF-7 cancer cell lines. Among the tested hybrids, compounds **37a–c** (Figure 7) displayed the highest antiproliferative activity against the tested cell lines. In the case of the normal WI-38 cell line, most of the synthesised compounds displayed IC_50_ values comparable to doxorubicin, indicating a favourable safety profile. An enzymatic assay was performed to evaluate the potential effect of the compounds on EGFR and CDK2. Compound **37c** showed the most potent effect against EGFR (IC_50_ = 0.12 µM) and CDK-2 (IC_50_ = 0.18 µM) (Table 7) when compared to Erlotinib for EGFR (IC_50_ = 0.15 µM) and roscovitine for CDK-2 (IC_50_ = 0.42 µM). Docking simulations were performed on active sites of EGFR (PDB: 1M17 and CDK-2 (PDB: 3DDQ). Compound **37c** showed favourable docking interactions with both EGFR and CDK-2, with strong binding energies and critical hydrogen-bonding patterns like the reference inhibitors, supporting its potential as a promising dual kinase inhibitor [65].

A series of novel triazole-tethered acridinedione hybrids was synthesised and evaluated for their biological activity against several breast cancer cell lines [90]. Among the derivatives, **38a** (Figure 7) displayed a significant anticancer activity against most of the cell lines. CDK inhibition assay indicated that **38a** and **38b** (Figure 7) displayed a strong preference for targeting CDK4/6 complexes. However, this selectivity pattern was not detected in the MDA-MB468 cell line. Apoptotic evaluation revealed that compound **38a** induced apoptosis in most of the tested cell lines; however, the MDA-MB468 cells remained largely resistant, showing no significant apoptotic response even at the maximum tested concentration (2 × IC_50_). In vivo evaluation indicated that compound **38a** displayed favourable pharmacokinetic properties in male albino mice. Docking analysis revealed that compound **38a** displayed several key interactions within the active sites of both CDK4 and CDK6. For CDK4, the compound formed hydrogen bonds with Arg37, Leu34, and Phe71, while for CDK6, it established hydrogen bonds with Arg245 and Glu189. These interactions closely mimic those of palbociclib, supporting the potential of **38a** as a dual CDK4/6 inhibitor [90].

**Table 7:** CDK inhibitory activity (IC_50_) of triazole-based compounds compared with a reference drug. Values are expressed in μM or nM.

Compound No.	CDK Inhibitory Activity		Reference
	(IC_50_)	Reference Drug	
	CDK2		[84]
**31**	87.9 nM	Roscovitine 140 nM	
	CDK9		[88]
**35a**	3.37 nM	AZD5438 18.25 nM	
**35b**	1.32 nM		
**35c**	2.90 nM		
	CDK2		
**37a**	0.33 µM	Roscovitine 0.42 µM	[65]
**37b**	0.19 µM		
**37c**	0.12 µM		

## 4. Benzimidazole–Triazole Hybrids for Protein Kinase Inhibition

Given the individual pharmacological importance of both benzimidazole and triazole scaffolds, researchers have explored their hybridisation to combine the biological advantages of each moiety. The fusion of these two heterocycles often enhances binding affinity, improves selectivity toward kinase domains, and contributes to better pharmacokinetic and metabolic stability [91]. In such hybrids, the benzimidazole ring typically serves as a core pharmacophore capable of anchoring within the hinge region of kinases. In contrast, the triazole ring acts as a versatile linker or substituent that facilitates additional hydrogen bonding or π–π stacking interactions within the active site. Consequently, benzimidazole–triazole hybrids have emerged as a promising class of kinase inhibitors with broad anticancer potential, as reflected in several recent studies [65,90,91,92,93].

### 4.1. Single Targeting

**Figure 8:**
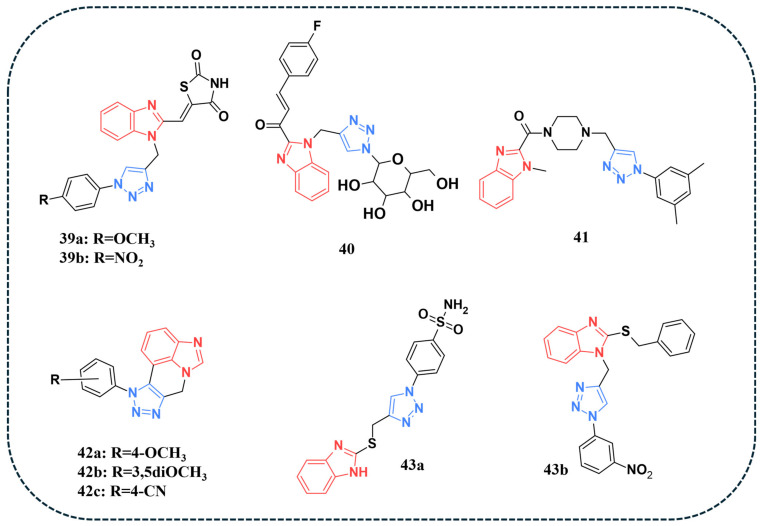
Benzimidazole–triazole hybrids, as reported in the literature and discussed in this review; compound codes are assigned in this review for consistency.

Vanaparthy et al. reported the synthesis of a novel series of benzimidazole–triazole analogues and evaluated the EGFR targeting capabilities of the compounds. MTT assays were performed on MCF-7, MDA-MB-468, and MDA-MB-231 cell lines. Several compounds showed promising results against the tested cancer cell lines, with low cytotoxicity against the normal breast cancer cell line. The EGFR inhibitory assay revealed that compounds **39a** and **39b** (Figure 8) showed the strongest inhibitory activity against EGFR, with IC_50_ values of 0.15 and 0.21 µM, respectively, surpassing the reference drug Erlotinib (IC_50_ = 0.42 µM) (Table 8). Additionally, several compounds were docked into the EGFR active site (PDB: 4HJO). The results showed that compound **39a** displayed the best binding energy (−11.04 kcal/mol), forming hydrogen bonds with Lys721, Cys751, and Phe832 [94].

Srour and co-authors designed and synthesised a panel of benzimidazole–triazole analogues bearing glycoconjugates and evaluated their anticancer properties against HepG-2, MCF-7, and HCT-116 cancer cell lines [95]. Several compounds demonstrated significant IC_50_ values against the tested cell lines and were further evaluated for their anti-EGFR activity. Compound **40** (Figure 8) displayed the best IC_50_ against EGFR (IC_50_ = 0.069 µM) (Table 8). Docking simulations were performed within the EGFR binding site (PDB: 4HJO). Compound **40** exhibited the best binding free energy score (−12.69 kcal/mol) and demonstrated strong EGFR inhibition (IC_50_ = 0.069 µM; ΔG = −12.69 kcal/mol). Its carbonyl group formed an H-bond with Thr766, while the triazole ring formed an H-bond with Met769, mimicking the hinge-binding role of Erlotinib’s quinazoline ring. The benzimidazole ring engaged in a π–π-sulfur interaction with Cys773, and the glycoside moiety formed H-bonds with Met769 and Val821 [95].

Benzimidazole and triazole scaffolds were also explored in a study by Pinnoju et al. [96], who synthesised novel benzimidazole–piperazine hybrids bearing a 1,2,3-triazole moiety and screened them against MDA-MB-231 and MCF-7 cell lines. Several compounds displayed significant potency against the cell lines, with compound **41** (Figure 8) displaying superior inhibitory activity within both cell lines (IC_50_ = 2.02 and 3.03 µg/mL, respectively). EGFR inhibitory activity was evaluated for several compounds, and the results indicated that compound **41** possessed the highest inhibition for EGFR, with an IC_50_ of 0.19 µM (Table 8). Docking simulations were performed on the EGFR binding site (PDB: 4HJO) and revealed that compound **41** displayed the highest binding energy (−10.33 kcal/mol), forming two hydrogen bonds with MET769 [96].

Novel benzimidazole–triazole hybrids were synthesised and examined for their antiproliferative properties against MCF-7 and MDA-MB-468 cell lines [97]. An MTT assay revealed that several compounds exhibited strong IC_50_ values against the tested cell lines. EGFR inhibitory activity was assessed, and **42a** and **42b** (Figure 8) demonstrated the best IC_50_ values (0.86 μM and 0.89 0.06 μM, respectively) (Table 8). Docking analysis of several compounds displayed comparable results, with **42c** exhibiting the most favourable binding energy (−8.22 kcal/mol), forming hydrogen bonding with ALA847, and π−cation and π-π-stacking with PHE699 [97].

Ahmed et al. designed a novel series of benzimidazole–triazole hybrids and tested their anti-cancerous activity against multiple cell lines (93). Among the tested compounds, **43a** and **43b** (Figure 8) demonstrated the best average IC50 values of 29 and 25 nM, respectively. Kinase inhibition assay revealed that compounds **43a** and **43b** were the most potent against EGFR, surpassing the reference drug Erlotinib (Table 8). Further assessment in the MCF-7 cell line indicated that both compounds displayed a significant rise in Bax expression, a protein that promotes apoptosis. Additionally, a pronounced reduction in Bcl-2 was reported. Molecular docking studies were performed on EGFR in complex with the anilinoquinazoline inhibitor Erlotinib (PDB: 1M17). Compound 43a showed a docking score of −7.41 kcal/mol, forming key interactions with Met769 and Asp831 along with a distinctive sulfonamide H-bond to Leu694. In comparison, compound 43b achieved a score of −8.17 kcal/mol, stabilised by water-bridged H-bonds with Thr766 and Pro770, in addition to an interaction with Leu820. For comparison, Erlotinib displayed a docking score of −11.80 kcal/mol [93].

**Table 8:** EGFR inhibitory activity (IC_50_) of benzimidazole–triazole hybrid compounds compared with the reference drug Erlotinib. Values are expressed in μM or nM.

Compound No.	EGFR Inhibitory Activity		Reference
	(IC_50_)	Reference Drug (Erlotinib)	
**39a**	0.15 µM	0.42 µM	[94]
**39b**	0.21 µM		
**40**	0.069 µM	0.048 µM	[95]
**41**	0.19 µM	0.40 µM	[96]
**42a**	0.86 µM	1.26 µM	[97]
**42b**	0.89 µM		
**42c**	2.38 µM		
**43a**	78 nM	80 nM	[93]
**43b**	73 nM		

### 4.2. Multi-Targeting

Benzimidazole–triazole hybrids are well recognised for their strong affinity toward EGFR, with numerous studies demonstrating their potency as EGFR inhibitors [92,93,95,96,98]. Recent studies have exploited the multi-kinase inhibition strategies to overcome the limitations and resistance commonly associated with single-target therapies. Building upon the well-established EGFR-directed activity, a few recent investigations have explored benzimidazole–triazole derivatives capable of targeting EGFR together with other kinases, representing the early development of this promising approach in anticancer research [92,99,100].

**Figure 9:**
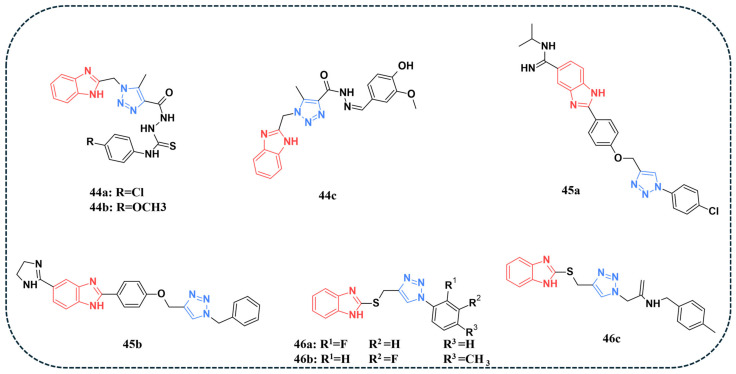
Benzimidazole–triazole hybrids targeting multiple kinases, as reported in the literature and discussed in this review; compound codes are assigned in this review for consistency.

A novel series of benzimidazole–triazole analogues was synthesised and evaluated for their anticancer potential against HepG-2, HCT-116, MCF-7, and HeLa cancer cell lines [92]. Among the tested derivatives, compounds **44a**, **44b**, and **44c** (Figure 9) demonstrated the most favourable IC_50_ values. While **44b** demonstrated significant activity only against MCF-7 and HeLa cell lines, compounds **44a** and **44c** exhibited consistent activity across all four cell lines. Against the normal WI-38 cell line, compounds **44a** and **44c** displayed significantly lower toxicity, with IC_50_ values of 37.16 and 43.28 µM, respectively, indicating a markedly enhanced safety profile compared to doxorubicin (IC_50_ = 6.72 µM). To test the multi-targeting properties of compounds **44a** and **44c**, enzyme inhibition studies were carried out against EGFR, VEGFR-2, and Topo II (Table 9). Although both compounds exhibited strong activity with favourable IC_50_ values against EGFR, VEGFR-2, and Topo II, compound **44a** demonstrated marginally better IC_50_ values compared to **44c**. However, when compared with the reference drugs, gefitinib showed higher IC_50_ values than **44a** in inhibiting EGFR, and sorafenib was more potent than **44a** against VEGFR-2. Interestingly, for Topo II, compound **44a** outperformed doxorubicin, exhibiting a lower IC_50_ value. Even though gefitinib and sorafenib showed slightly better IC_50_ values, the differences were very small. Hence, compound **44a** can still be considered a strong candidate with comparable activity. Docking simulations were performed on Top II (PDB: 1ZXM), VEGFR-2 (PDB: 2OH4), and EGFR (PDB: 2J6M) active sites. Compound **44a** consistently showed better docking scores and stronger key interactions than **44c**, particularly due to its carbothioamide linker and ability to form stabilising H-bonds. Compound **44c** interacted with the targets but with weaker stabilisation and less optimal orientation [92].

Bistrovic and co-authors synthesised a panel of benzimidazole–triazole hybrids and tested their antiproliferative effects against multiple cell lines [101]. An MTT assay revealed that several benzimidazole–triazole derivatives exhibited potent antiproliferative effects, with compound **45a** showing remarkable activity against A549 lung cancer cells (IC_50_ = 0.06 µM). Annexin V test revealed that both compounds **45a** and **45b** (Figure 9) triggered apoptosis in A549 lung cancer cells, with compound **45a** serving as the stronger apoptosis inducer. Western blot analysis demonstrated that compound **45b** reduced levels of CDK9/cyclin T1 in A549 cells, which is also associated with a reduction in phospho-p53 (Ser15) levels. Furthermore, the authors hypothesised that the downregulation of p53 activity in A549 cells by compound **45b** might be attributed to the inhibition of several kinases, including TGM2, CDK9, and p38 MAPK. In contrast, compound **45a** did not markedly affect CDK9 or TGM2 but moderately reduced SK1 expression along with a pronounced decrease in p38 MAPK activity. Docking results showed that both compounds **45a** and **45b** were potent p38 MAPK inhibitors, with compound **45b** exhibiting a stronger and more stable binding mode. This was attributed to additional hydrogen bonds with hinge residue Met109 and polar residues (Glu71, His148, and Asp168), as well as favourable π–π stacking with Phe169. This explains the lower predicted binding energy of compound **45b** and aligns with biological assays showing that this compound induced significant downregulation of phospho-p38 MAPK levels in the lung cancer cell line [101].

Benzimidazole–triazole analogues were synthesised for both antimicrobial and anticancer applications [102]. For anticancer evaluation, MTT assays were performed utilising HepG-2, MCF-7, HCT-116, and A549 cell lines. Compounds **46a**, **46b**, and **46c** (Figure 9) demonstrated favourable IC_50_ against the tested cell lines and were further evaluated for their kinase inhibitory activity. Compound **46c** demonstrated the best inhibition for EGFR-TK^WT^, with an IC_50_ value of 0.32 ng/mL (Table 9) and was also found to downregulate EGFR-T790 and VEGFR-2, thereby hindering angiogenesis. Molecular docking of **46c** into the active site of EGFR^T790^ (PDB: 5FEE) and EGFR^WT^ (PDB: 4HJO) demonstrated that **46c** fit well within the active site, adopting a binding mode like the co-crystalised inhibitor [102].

**Table 9:** Multi-kinase inhibitory activity (IC_50_) of benzimidazole–triazole hybrid compounds. Values are expressed in μM or ng/mL.

Compound No.	Kinase Inhibitory Assay			Reference
	IC_50_			
	Targets			[92]
	EGFR	VEGFR-2	Topo II	
**44a**	0.086 µM	0.107 µM	2.52 µM	
**44c**	0.131 µM	0.229 µM	8.37 µM	
Gefitinib	0.052 µM	-	-	
Sorafenib	-	0.048 µM	-	
Doxorubicin	-	-	3.62 µM	
	Targets			[102]
	EGFR-TK^WT^	EGFR^T790^	VEGFR-2	
**46a**	0.51 ng/mL	17.63 ng/mL	1.79 ng/mL	
**46b**	1.25 ng/mL	35.5 ng/mL	2.55 ng/mL	
**46c**	0.32 ng/mL	10.05 ng/mL	1.27 ng/mL	
Reference drug	10.01 ng/mL	75.58 ng/mL	3.31 ng/mL	

## 5. Structure–Activity Relationships

**Figure 10:**
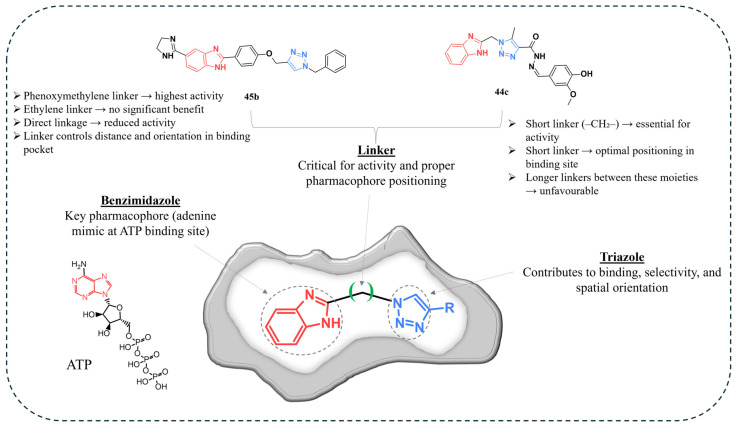
Illustration of benzimidazole–triazole hybrids showing hinge binding and linker-controlled spatial arrangement within the kinase active site [92,101].

Benzimidazole and triazole play distinct roles in kinase inhibition. Benzimidazole structurally resembles purine (adenine), enabling it to mimic adenine-like interactions and bind within the ATP-binding site through hydrogen bonding with hinge residues (Figure 10) [22,103]. This enables benzimidazole-containing compounds to interact with the kinase hinge region, contributing significantly to binding affinity and potency.

In contrast, the triazole moiety contributes directly to binding interactions in addition to its role as a linker. 1,2,3-Triazoles can form hydrogen-bonding and dipole–dipole interactions with biological targets, allowing them to stabilise ligand–protein complexes [37]. The nitrogen-rich structure enables participation as hydrogen-bond acceptors within enzyme active sites, while the N1 and N2 atoms can act as key interaction points required for inhibition.

Triazoles can also function as bio-isosteric substitutes for peptide bonds, preserving favourable geometry while introducing rigidity into the molecular framework [104]. This supports optimal spatial arrangement of pharmacophoric groups and enables interactions across multiple regions of the kinase active site, thereby contributing to selectivity.

The multitarget activity observed in benzimidazole–triazole hybrids can therefore be attributed to the integration of these complementary features, including a hinge-binding benzimidazole core and a triazole moiety functioning as both a linker and an active interaction site. Together, these structural elements enable simultaneous engagement with multiple kinase targets, supporting the development of compounds with broad-spectrum anticancer activity.

Moreover, the nature and length of the linker between benzimidazole and triazole play a critical role in determining biological activity, as variations in linker type significantly influence antiproliferative potency, target interaction, and overall binding within the kinase active site.

## 6. Conclusions and Perspectives

Benzimidazole and triazole scaffolds have emerged as highly promising candidates in anticancer drug discovery owing to their unique structural versatility, favourable pharmacokinetic properties, and broad biological activity. Hybridisation of these two heterocycles into a single compound not only integrates the advantages of both scaffolds but also demonstrates enhanced biological efficacy, including the ability to modulate multiple pathways simultaneously. This multi-targeting approach is especially valuable in overcoming the limitations of conventional single-target anti-cancer therapies, such as drug resistance and pathway compensation, ultimately offering more robust and durable anticancer responses.

However, multi-target activity may also introduce potential limitations, including reduced selectivity and an increased risk of off-target toxicity, emphasising the importance of careful structural optimisation. The contribution of the benzimidazole moiety appears to depend strongly on its spatial exposure within the hybrid structure. When positioned terminally, the nitrogen atoms can participate in hydrogen bonding while the aromatic system engages in π–π stacking interactions, directly contributing to targeted binding. In contrast, when embedded within the molecular backbone, benzimidazole mainly acts as a conformational scaffold that controls rigidity and geometry rather than forming strong interactions with the biological target. These findings indicate that pharmacophore accessibility, rather than presence, determines biological activity.

In this context, structural parameters controlling the spatial relationship between benzimidazole and triazole remain insufficiently explored. A single investigation evaluating the linker between both moieties indicated that excessive shortening of the distance is detrimental to activity [101], whereas simple spacer extension did not improve efficacy, suggesting the existence of an optimal pharmacophore orientation. Therefore, studies examining linker length, rigidity, and functionality are required to clarify SAR and to guide the rational design of safer, more selective benzimidazole–triazole anticancer agents.

## Data Availability

No new data were created or analysed in this study. Data sharing is not applicable to this article.
